# Domestic Violence during Pregnancy and Mental Health: Exploratory Study in Primary Health Centers in Peñalolén

**DOI:** 10.5402/2011/265817

**Published:** 2010-10-28

**Authors:** R. Carla Crempien, Graciela Rojas, Patricio Cumsille, M. Camila Oda

**Affiliations:** ^1^Psychotherapy Research Doctoral Program, Departamento de Psicología, Pontificia Universidad Católica de Chile, Avenida Vicuña Mackenna 4860, Macul, 8320000 Santiago, Chile; ^2^Hospital Clínico Facultad de Medicina, Universidad de Chile, 8320000 Santiago, Chile; ^3^Departamento de Psicología, Pontificia Universidad Católica de Chile, 8320000 Santiago, Chile; ^4^Clinical and Health Psychology Master Program, Universidad de Barcelona, 08007 Barcelona, Spain

## Abstract

*Objective*. To determine the prevalence of domestic violence in a sample of pregnant women attending Primary Health Centers in Peñalolén (Peñalolén is a low income district in the Metropolitan Region in Santiago de Chile.), to explore risk facts for domestic violence during pregnancy, and to establish associations with their psychological health. *Method and Materials*. 256 pregnant women were assessed with a domestic violence screening and a questionnaire on mental symptoms. Frequency and correlations analysis were developed. *Results*. 5, 9% of the participants reported physical violence during current pregnancy. Emotional violence ascended to 30, 1% of the cases. Main risk facts found were as follows: having suffered violence along lifetime and physical violence during the last year. Anxiety and depressive symptoms positively correlated to domestic violence during pregnancy, but also to previous domestic violence experiences. *Conclusions*. Domestic violence during pregnancy is a prevalent problem and domestic violence history constitutes an alert to its occurrence. Positive and significant association to psychological disturbances suggests the need to detect it early during antenatal care.

## 1. Introduction

The context of intimacy typically associated with domestic violence often keeps women from spontaneously externalizing this problem.

A large number of studies have dealt with the issue of domestic violence during pregnancy, focusing on its prevalence, risk factors, and consequences, and on the role of health teams in its detection, prevention, and treatment [1–3].

The prevalence rates of domestic violence during pregnancy range between 4.8% and 8.1% in most international studies [3]. In Chile, in the Metropolitan and Araucanía regions, 10% of the women who experienced domestic violence reported having been hit by their partners during pregnancy [4].

Some of the problems associated to domestic violence during pregnancy are femicide, abortion, infant mortality, obstetric complications, infant morbidity, and mental health problems for the mother, as well as risk behaviors, and deterioration of mother-child attachment [5–12].

Although the evidence is controversial to suggest that it is a particularly risky period, it has been observed that pregnancy does not protect women from intimate partner violence [1, 5].

The association between violence during pregnancy and adverse mother and perinatal outcomes stresses the need to identify this situation in antenatal care [6, 13]. This would help pregnant women subjected to violence to speak out, escape the emotional and social isolation which they commonly experience, and receive specialized help.

The objectives for this study are (1) to evaluate the magnitude of the problem of domestic violence during pregnancy in a sample of pregnant women who attend primary health centers in Peñalolén for their antenatal care; (2) to explore the risk factors of domestic violence during pregnancy in this group; (3) to establish associations between domestic violence and mental symptoms during pregnancy.

## 2. Material and Methods

This study is nonexperimental, exploratory, descriptive, and correlational.

### 2.1. Sample and Data Collection

A convenience sample was used in this study. 256 pregnant women attending Peñalolén health centers for antenatal care, between September 2006 and January 2007, were asked to respond to a set of instruments composed by a screening questionnaire for pregnancy violence based on the Abuse Assessment Screen [14], the Goldberg's General Health Questionnaire for psychic discomfort symptoms [15], and a form to gather sociodemographic data.

The data were collected by 12 qualified interviewers, psychology students, who received training to apply the instruments and to respect the ethical conditions of their task: privacy and comfort during the application of the instruments, informed consent, and confidentiality.

The positive cases for domestic violence were informed via written reports, and with the women's informed consent, to the head midwives of the relevant units, for the future mothers to receive support or to be referred to a specialized intervention.

The data collection procedure was the following: in the waiting rooms of midwives' offices, during pregnant women's antenatal care, they were invited to participate in the study, received information about its objectives, the procedures involved, and the confidentiality of the data gathered. All the pregnant women who agreed to participate were included; no exclusion criteria were set.

 The sample size was determined in order to have appropriate statistical power for the expected effect size. Conservatively, we estimated that for *r* = 0.2, alpha = 0.01 to have power = 0.8 we needed 244 cases.

### 2.2. Instruments

#### 2.2.1. Screening of Domestic Violence during Pregnancy

This instrument was based on the Abuse Assessment Screen (AAS), developed by McFarlane et al. [14] to detect domestic violence during pregnancy. It is a short questionnaire, easy to apply, validated, and widely used in international studies. The final questionnaire included 5 questions from the original instrument: occurrence of physical and/or emotional violence in the woman's life; occurrence of physical violence exerted by the intimate partner, ex-partner, or a relative during the last year; occurrence of sexual violence exerted by the woman's intimate partner or ex-partner in the last year; if the woman fears her intimate partner, ex-partner or a relative, the occurrence of physical violence during current pregnancy. A sixth question was added to establish the occurrence of emotional violence exerted by the woman's intimate partner, ex-partner, or a relative during pregnancy. The questions about physical and emotional violence include a description and examples of the manifestations of such abuse. The question about sexual violence refers to whether the woman has been forced to engage in sexual acts against her will.

#### 2.2.2. Goldberg's General Health Questionnaire [15], in its 12-Question Form (GHQ-12)

It evaluates, with an adequate degree of reliability and validity, a person's psychological discomfort or emotional symptoms in the last month, and it is considered an indicator of symptomatology associated to mental disorders. It comprises twelve questions with four alternatives each, two of them scoring zero points (0) and the other two scoring one (1), which results in a possible minimum score of zero (0) and a maximum of twelve [12]. It has been validated and used in Chile. A score equal to or higher than five (5) is regarded as suggesting risk of emotional pathology [16].

#### 2.2.3. Sociodemographic Form

 The form included questions about the woman's age, marital status, educational level, occupation, family members with whom she lives, number of people who live with her, number of children, weeks of pregnancy, gestational age at the beginning of her antenatal care, presence of pregnancy pathologies, referral to a program for risky pregnancies, and nutritional state. Women subjected to violence of some sort during pregnancy were asked about their relationship with their assailant and his use of alcohol and/or illegal drugs.

#### 2.2.4. Data Analysis

 A series of statistical tests were conducted to analyze the data. Frequency analyses were developed to establish the prevalence of domestic violence and the presence of mental symptoms during pregnancy. To explore significant associations with the control variables (age, number of people in the household, weeks of pregnancy, and number of children), a one-factor Anova was conducted; to observe the relation between domestic violence during pregnancy and the rest of the variables (nominal), non-parametric correlation tests were used (Chi-square, Cramer's V, and Phi).

## 3. Results

### 3.1. Statistical-Descriptive

The sample evaluated was comprised of 256 cases, in which the average age was 25 years (SD = 6.65); the average number of pregnancy weeks was 28.2 (SD = 8.06); the average number of people who lived with the women sampled was 4.5 (SD = 2.39), and the average number of children was 0.9 (SD = 1.06).

Most participants (64.1%) were housewives, 30.9% were students or had a stable job, and 5% had sporadic jobs. Regarding their educational level, most women had complete high school studies (45.3%), or incomplete ones (27.3%); 10.2% had completed elementary school, 8.6% had not, and the same percentage of women (8.6%) had completed their technical or university studies.

Concerning their marital state, 43% of the women sampled lived with their couples, not being married to them, 29% were single, and 28% were married.

Most of the participants started their ante natal care with less than 12 weeks of gestation (79%), while 21% did so after 12 weeks. With respect to their nutritional state, 56.6% of the women had a normal weight; 27% were overweight, 9.8% were obese, and 6.6% were below their normal weight. 82.4% of the women sampled were not part of the program to control the risk of a premature birth, whereas 82.8% had no obstetric complications.

In the evaluation of domestic violence, a high percentage of the pregnant women (42.2%) reported antecedents of domestic violence in their lives (history of violence); 14.5% reported having suffered physical violence in the last year, and 3.5% mentioned having suffered sexual violence in the last year. 11.3% of the sample pointed out that they feared their intimate partner, ex-partner, or a relative.

Regarding the occurrence of domestic violence in their current pregnancy, 5.9% reported having suffered physical violence and 30.1% reported the presence of emotional violence (see Table 1).

All the women who reported physical violence in their pregnancy also stated that they had suffered emotional violence; thus, 30.1% of the sample reported having suffered violence of some sort during the current gestational process. 

When violence was reported (either physical, emotional, or both), it was exerted by the woman's intimate partner in 53.2% of the cases, by another family member (31.2%), by the woman's former partner (11.7%), or by her partner and a relative (3.9%). 33.8% of the assailants were heavy or problem drinkers, according to the pregnant women, while 15.6% of them used illegal drugs. 

With regard to the assessment of mental discomfort, 42.2% of the women evaluated had a GHQ-12 score classified as positive, in other words, they presented anxious or depressive mental symptoms.

### 3.2. Statistical Tests to Establish Associations between the Variables Studied

To evaluate the presence of a connection between domestic violence during pregnancy and the other variables observed, a series of statistical tests were conducted. To explore whether there was a significant connection with the control variables (age, number of people in the household, weeks of pregnancy, and number of children), a one-factor Anova was conducted; to observe the relation between the presence of domestic violence during pregnancy and the rest of the variables (nominal), non-parametric correlation tests were used, such as Chi-square, Cramer's V, and Phi, which revealed the following significant associations. 

A statistically significant, direct, and moderate correlation was found between violence during pregnancy and a woman's history of domestic violence (*r* = 0.46, *P* = .000) (see Table 2), that is to say, the presence of antecedents of domestic violence at any point of a woman's life is associated to domestic violence during pregnancy.

There is a statistically significant, direct, and low correlation between domestic violence during pregnancy and physical violence during the last year (*r* = 0.38, *P* = .000) (see Table 3); also, a statistically significant, direct, and low correlation was found between violence during pregnancy and fear of the partner, ex-partner, or a relative (*r* = 0.30, *P* = .000).

The GHQ-12 results obtained, that is, the presence of psychological discomfort symptoms (5 or more points), also reveals a direct and low correlation with the occurrence of domestic violence during pregnancy (*r* = 0.32, *P* = .000).

Afterwards, the relation between participants' GHQ-12 score and the rest of the variables was tested, in order to observe if a positive test score could be predicted based on the variables assessed. The following significant associations were observed.

There is a direct and low correlation between GHQ-12 and a history of domestic violence (*r* = 0.247, *P* = .000). There is a direct and low correlation between GHQ-12 and the presence of physical domestic violence in the last year (*r* = 0.256, *P* = .000). There is a direct and low correlation between GHQ-12 and the presence of emotional domestic violence during pregnancy (*r* = 0.32, *P* = .000). In brief, the presence of psychic discomfort symptoms in pregnant women displays a positive, but low, connection not only with the occurrence of all forms of violence during pregnancy, but also with physical violence in the last year and with all sorts of domestic violence in their lifetime (see Table 4).

## 4. Discussion

Within the sample, the prevalence of physical domestic violence during pregnancy reached 5.9%, which is comparable to that observed in other settings [6, 17]. This finding is consistent with studies which have shown that pregnancy does not protect women from violence, and that violence is likely to continue even when it is present [1].

Most of previous studies do not include the emotional violence variable; if considered, it makes the figure climb to 30.1% in the sample. As this variable shows a positive and significant association with the presence of psychological discomfort symptoms in pregnant women, it seems relevant to include the assessment of emotional violence during pregnancy.

The variables with a significant association to violence during pregnancy are: history of domestic violence, physical violence in the last year, the presence of fear, and a positive GHQ-12 score. These elements in a pregnant woman may suggest that she is experiencing domestic violence in her pregnancy, but at this point a limitation of the current study must be pointed out regarding the fact that statistical analysis were not adjusted for potential confounding variables. Nevertheless, a woman's history of domestic violence and the presence of physical violence in the last year should be considered an alert to domestic violence during pregnancy and, therefore, these women should be carefully followed up during pregnancy. 

A previous Chilean study showed a strong association between women mental disorders, family violence and socioeconomic factors. The combination of these variables increases the association and this is particularly evident when there is a history of family violence [18]. In our study, positive and significant associations were found between the presence of mental symptoms (psychcological discomfort) during pregnancy and a history of domestic violence, physical violence in the previous year, domestic violence in any of its forms during pregnancy, and specifically emotional violence during pregnancy. These findings clearly show the connection between violence and pregnant women's mental health, and point to two relevant aspects: the importance of an early detection of violence, and alertness regarding the possibility of finding violence during the pregnancy in women with depressive and/or anxiety symptoms.

Regarding the screening of violence during pregnancy, it can be advanced that, for pregnant women subjected to violence, healthcare workers can be an important point of contact with the public services capable of providing support and information [10, 19]; also, there is evidence that brief and direct questionnaires produce better data than normal obstetric interviews, which do not include direct questions about domestic violence [9]. The results presented in this study with pregnant women from Peñalolén suggest the importance of addressing this issue for preventing any adverse consequences in pregnant women and their children.

## Figures and Tables

**Table 1:** Domestic violence during pregnancy.*

*N* = 256	Frequency	Percentage
Physical abuse		
Yes	15	5, 9%
No	241	94, 1%

Emotional abuse		
Yes	77	30, 1%
No	179	69, 9%

*Pregnant women attending antenatal care at Primary Health Centers in Peñalolen (Chile, 2006-2007).

**Table 2:** Domestic violence during pregnancy and history of violence.*

*N* = 256	Value	Significance
Phi	.457	.000
Cramer V	.457	.000
Contingence Coefficient	.416	.000
Pearson R	.457	.000(c)
Spearman Correlation	.457	.000(c)

*Pregnant women attending antenatal care at Primary Health Centers in Peñalolen (Chile, 2006-2007).

**Table 3:** Domestic violence during pregnancy and physical violence during the last year.*

*N* = 256	Value	Significance
Phi	.384	.000
Cramer V	.384	.000
Contingence Coefficient	.359	.000
Pearson R	.384	.000(c)
Spearman Correlation	.384	.000(c)

*Pregnant women attending antenatal care at Primary Health Centers in Peñalolen (Chile, 2006-2007).

**Table 4:** Mental symptoms (GHQ-12) and domestic violence.*

*N* = 256	Value	Significance
Violence during pregnancy		
Phi	.319	.000
Cramer V	.319	.000

History of domestic violence		
Phi	.247	.000
Cramer V	.247	.000

*Pregnant women attending antenatal care at Primary Health Centers in Peñalolen (Chile, 2006-2007).
